# Importance of cerebrospinal fluid investigation during dengue infection in Brazilian Amazonia Region

**DOI:** 10.1590/0074-02760180450

**Published:** 2018-12-10

**Authors:** Michele de Souza Bastos, Valquiria do Carmo Alves Martins, Natália Lessa da Silva, Samya Jezine, Sérgio Pinto, Valderjane Aprigio, Rossicleia Lins Monte, Silvio Fragoso, Marzia Puccioni-Sohler

**Affiliations:** 1 Fundação de Medicina Tropical Dr Heitor Vieira Dourado, Manaus, AM, Brasil; 2 Fundação Centro de Controle de Oncologia do Estado do Amazonas, Manaus, AM, Brasil; 3 Universidade Federal do Estado do Rio de Janeiro/Universidade Federal do Rio de Janeiro, Rio de Janeiro, RJ, Brasil

**Keywords:** flavivirus, cerebrospinal fluid, dengue virus, immunoglobulin IgM, meningitis, encephalitis

## Abstract

BACKGROUND Amazon, the largest tropical forest of the world, has suffered from dengue outbreaks since 1998. Cerebrospinal fluid (CSF) of patients, from Amazonas state, suspected of central nervous system (CNS) viral infection was studied using molecular and immunological methods. OBJECTIVE To evaluate the importance of CSF investigation in patients with acute dengue virus (DENV) infection of CNS. METHODS CSF samples of 700 patients were analysed by reverse transcription polymerase chain reaction (RT-PCR) to detect the presence of dengue virus (DENV) RNA and by enzyme-linked immunosorbent assay (ELISA) to detect presence of DENV specific IgM. FINDINGS DENV infection was detected in 4.3% of the CSF samples; 85.7% (24/28) by DENV IgM and 14.3% (4/28) by viral RNA. DENV detected by viral RNA were to be found serotypes DENV-2 (three patients) and DENV-1 (one patient). The neurological diagnosis in patients CNS infection of DENV included encephalitis (10), meningoencephalitis (10), meningitis (6), acute myelitis (1), and encephalomyelitis (1). The majority (89.3%) had intrathecal inflammation: pleocytosis, hyperproteinorrachia and DENV IgM antibodies. Hypoglycorrhachia and/or high levels of lactate in CSF were found in 36% of the patients. Co-infection (CMV, HIV, EBV, and/or *Mycobacterium tuberculosis*) was observed in eight (28.6%) cases. CONCLUSIONS We found intense inflammatory CSF that is unusual in CNS disorders caused by dengue infection. It may be due co-infections or the immunogenetic background of the local Amerindian Brazilian population. CSF examination is an important diagnostic support tool for neurological dengue diagnosis.

Dengue virus (DENV) is a sense stranded RNA virus, belonging to genus *Flavivirus* a member of *Flaviviridae* family, and has four distinct serotypes (DENV 1-4). It is a vector-borne disease mainly transmitted by the mosquito *Aedes aegypti*. Dengue is an endemic disease that causes about 50 to 100 million infections annually in more than 100 countries. Half of the world’s population is at risk of dengue infection.^1^

Amazonia is the largest tropical forest of the world and majority of it is located in Amazonas state, Brazil. The state has a population of almost three and half million people and has highest Amerindian ancestral influence observed in Brazil.^2^ Manaus is the largest city in the state. The population has suffered due to several dengue outbreaks with the introduction of DENV-1 in 1998, followed by DENV-2 in 2001, DENV-3 in 2002, and DENV-4 in 2008.^3^ Since then, severe cases of infection, mostly in children have increased with every outbreak.^4^ A new outbreak with simultaneous circulation of the four dengue serotypes started in Manaus in 2011. There was clear evidence of hyperendemicity with 40,000 cases of infection resulting in increased morbidity and mortality.^5^

Dengue infection is often oligosymptomatic, but may also lead to a wide range of clinical manifestations. These include warning signs like fever, rash, vomiting or can result in plasma leakage, haemorrhage, severe shock (DSS). Severe cases can also lead to organ involvement, including the liver and central nervous system (CNS), which can be potentially fatal.^1^ The main neurological manifestations of dengue consist of encephalitis, transverse myelitis, meningitis, Guillain Barré syndrome, and neuromyelitis optica.^6^ There are only few case reports or short series that discuss the laboratory characterisation of dengue infection in CNS. The use of virological tests in combination with cerebrospinal fluid (CSF) analysis may improve the sensitivity and specificity for neurological diagnosis.

In this study, we have evaluated the frequency of dengue infection in CSF samples of patients from a Brazilian population with strong indigenous ancestral influence,^2^ living in a endemic area for DENV (Manaus, Amazonas)^5^ ^,^ ^7^ and are suspected of viral infection of the CNS.

## SUBJECTS AND METHODS

*Study design, population and period* - A retrospective study was conducted at the Tropical Medicine Foundation (FMT-HVD). It is a tertiary public health institute and reference centre for infectious diseases and CSF analysis in the Amazonas state. All patients suspected of viral infections in CNS (acute meningitis, encephalitis, and myelitis) received treatment in the emergency department of FMT-HVD during period 2011-2017. Clinical data belonging to 700 patients and their catalogued CSF samples were studied for evidence of dengue infection. Some of them were found to be seropositive for HIV infection and were enrolled in the program for sexually transmitted infections (STIs) and HIV/AIDS at FMT-HVD.

*Suspected cases of neurological dengue infection and case definition* - The criteria for suspected viral infection of the CNS included neurological signs and symptoms, such as headache, fever, focal neurologic findings, and altered consciousness or cognition.^1^ ^,^ ^8^ Lumbar puncture to get CSF sample was performed on all of the patients on the day of admission. An aliquot of CSF was submitted for routine analysis: total and differential cell counts by cytosedimentation; determination of protein, glucose, and lactate by spectroscopy; and microbiological tests for bacteria and fungi (smear, culture, and latex).^9^ The criteria for samples to be considered as having dengue infection included positive result for the presence of cDNA by reverse transcription polymerase chain reaction (RT-PCR) or detection of IgM antibodies in CSF specific to DENV by enzyme-linked immunosorbent assay (ELISA).^1^ ^,^ ^8^

*Ethics* - This study was part of an ongoing nervous system viral infection surveillance program approved by the Ethical Review Board of the Fundação de Medicina Tropical Dr Heitor Vieira Dourado (FMT-HVD) # 43123315.2.0000.0005.

*Laboratory methods* - The CSF samples (diluted 1:5) were analysed for presence of IgM antibodies to DENV by an in house MAC-ELISA test kit.^10^ Diagnosis of dengue infection by RT-PCR was done using nucleic acids purified from 200 µL of CSF. The nucleic acids were purified by using the QIAmp viral RNA/DNA Mini Kit (QIAGEN, Valencia, CA), according to the manufacturers instruction. AccessQuick RT-PCR System kit (Promega, USA) was used for reverse transcription (RT) of viral RNA for cDNA synthesis. The DENV serotype was determined by using a semi-nested multiplex RT-PCR protocol as described elsewhere.^11^ Briefly, each cDNA was subjected to polymerase chain reaction (PCR) amplification with D1 and D2 primers. The sample which showed positive signal were subjected to a second round of amplifications by using the conserved forward primer D1 and DENV serotype-specific reverse primers (TS1, TS2, TS3, and TS4). The reaction mixture from first round of amplification was diluted by 1:100 and 1 µL was used as template.

*Nucleotide sequencing and phylogenetics* - Purification of the PCR product was done with one step pipetting to reduce the chances of cross contamination. Amplicons were treated with exonuclease I (20 U/µL) (New England BioLabs, USA) and shrimp alkaline phosphatase (1 U/µL) (Fermentas, USA). Purified amplicons were directly sequenced using the BigDye Terminator Cycle Sequencing Kit (Applied Biosystems, EUA), following manufacturer instructions on an automatic sequencer (ABI 3130L DNA Analyzer, Applied Biosystems, EUA). Nucleotide sequences were analysed by algorithms of the BioEdit Sequence Alignment software (Version 7.2.5).^12^ Nucleotide and putative amino acid sequences were compared to other sequences from GenBank for phylogenetic analysis by neighbour-joining method using the software MEGA7 X (Version 10.0.1) (Arizona State University, AZ, USA) with 1000 bootstrap replications.^13^ These sequences were deposited in GenBank database and can be accessed with the following accession numbers: 106-CNS-DENV-1 (MH397677); 108-CNS-DENV-2 (MH397678); 25-CNS-DENV-2 (MH397679); and 111-CNS-DENV-2 (MH397680).

*Screening of other viruses* - RT-PCR was used for screening presence of *St. Louis encephalitis virus* (SLEV), *Rocio virus* (ROCV), *Ilheus virus* (ILHV),^15^ *Zika virus*,^16^ *Chikungunya virus*,^17^ and *Enterovirus* ^18^ in cDNA samples obtained from CSF of patients. The CSF samples were also screened for *Herpes simplex virus* types 1 and 2 (HSV-1 and HSV-2), *Cytomegalovirus* (CMV), *varicella zoster virus* (VZV), and *Epstein-Barr virus* (EBV) using multiplex PCR protocol described elsewhere.^14^

## RESULTS

Molecular and immunological methods were used to analyse CSF from 700 patients suspected of DENV infection of CNS. Laboratory tests confirmed viral infection in 30 (4.3%) cases, presenting with acute neurological manifestations. Two patients were excluded from the study due to the lack of clinical information. Clinical and laboratory findings are shown in Table.

**Fig. 1:**
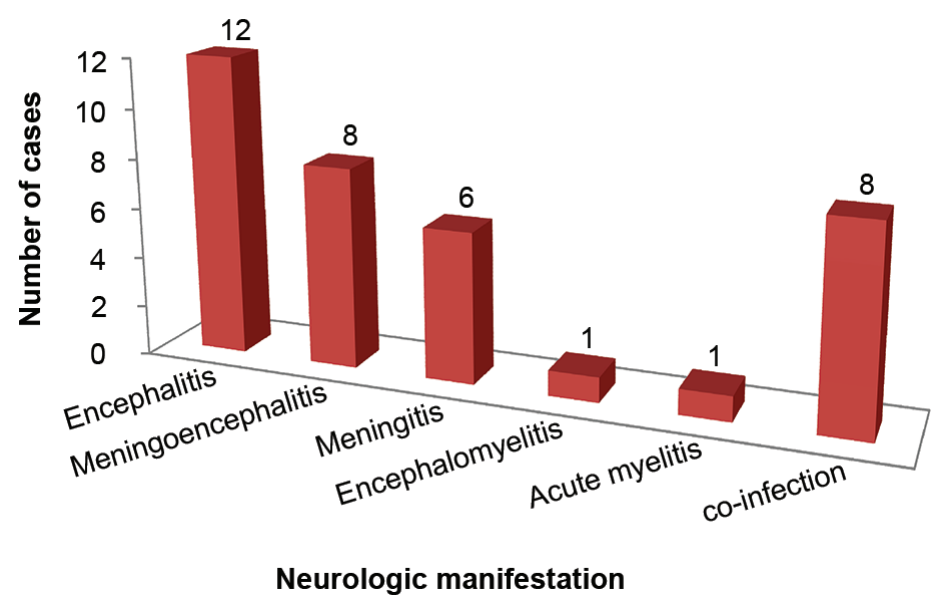
frequency of neurologic manifestations in 28 confirmed cases of central nervous system (CNS) infection by dengue virus (DENV).

The median age of the 28 patients was 28.5 years old (ranging from 4 to 66 years) with 7 out of 28 (25%) patients were ≤ 18 years old and 15/28 (53.6%) patients were male. Most patients (92.9%) lived in the city of Manaus and only two were from other municipalities within Amazonas state (Itacoatiara and Manacapuru). The patients with viral infection of CNS by DENV mainly presented with headache (64.3%), disturbed consciousness (57.1%), and fever (39.3%). A cutaneous rash was observed in five (17.8%) cases. Four (14.3%) patients had clinical history of acute dengue infection based on earlier serology diagnosis (specific IgM). The majority (71.4%) of patients developed encephalic manifestations (encephalitis, meningoencephalitis, or encephalomyelitis) (Fig. 1).

The CSF showed signs of inflammation with pleocytosis (> 4 cells/mm^3^) reported in 25 patients (89.2%, 25/28) with nine patients (32.1%, 9/28) being flagged (> 200 cells/mm^3^). These flagged cases included the five cases suspected of viral encephalitis / meningoencephalitis and four cases of meningitis. The median CSF white blood cell (WBC) count was 84.5 cells/mm^3^ (range 0-2048). Lymphocytic pleocytosis was predominant in the majority of cases (median percentage of lymphocytes 98.5%). Nineteen patients (67.8%) had hyperproteinorrachia (> 45 mg/dL), which was very pronounced in nine patients (47.4%, 9/19) and was in range of 100-500 mg/dL. The median level of protein in CSF was 68.3 mg/dL (range 7-483 mg/dL). The median levels for glucose in CSF was 60.5 mg/dL (range: 14-797 mg/dL) and lactate levels in CSF was 2.5 mmol/L (range: 0.7-6.34 mmol/L) in CSF.

**TABLE Individual:** clinical and laboratory features of the 28 patients with dengue virus (DENV) infection in central nervous system (CNS)

Nº	Age/Sex	Diagnosis	Signs and symptoms	Co-infection	WBC/mm^3^ (%)	Protein mg/dL	Glucose mg/dL	Lactate mmol/L	CSF IgM	PCR/CSF
1	20/M	Encephalitis	Headache, focal neurological signs	HIV	96 100% MNN	142	166	5.0	+	EBV
2	51/M	Encephalitis	Headache, disorientation		154 100% MNN	64	30	1.0	+	-
3	30/F	Encephalitis	Disorientation, focal neurological signs	HIV	6 100% MNN	101	39	1.6	+	-
4	27/F	Encephalitis	Decreased consciousness		0	22	83	0.8	+	-
5	9/M	Encephalitis	Headache, fever, disorientation		373 91% MNN 9% PMN	75.6	49	2.7	-	DENV-2
6	50/M	Encephalitis	Fever, headache, disorientation		384 100% MNN	123	48	1.9	+	-
7	35/M	Encephalitis	Headache, myalgia, neck stiffness	HIV	186 100% MNN	70	40	2.2	+	-
8	43/M	Encephalitis	Focal neurological signs	HIV	0	27	57	1.4	+	-
9	38/F	Meningoencephalitis	Focal neurological signs		1226 100% MNN	70	797	6.4	+	-
10	66/F	Meningoencephalitis	Fever, Focal neurological signs	HIV, MT	2048 95% MNN 5% PMN	311	40	6.3	+	-
11	4/M	Encephalitis	Focal neurological signs		23 100% MNN	84	40	1.6	+	-
12	32/F	Encephalitis	Headache,Paresthesia of lower limbs, urinary incontinence		30 100% MNN	32	77	1.9	+	-
13	42/M	Meningoencephalitis	Headache, vomiting, drowsiness	HIV	73 91% MNN 9% PMN	483	34	4.0	+	-
14	25/F	Meningoencephalitis	Headache, fever, neck stiffness		20 100% MNN	24	75	0.7	-	DENV-2
15	23/M	Meningoencephalitis	Fever, disorientation, left hemiparesis	HIV	170 99% MNN	32	59	2.7	+	-
16	39/M	Meningoencephalitis	Fever; headache, myalgia, decreased consciousness		60 100% MNN	128	63	2.0	+	-
17	40/F	Meningoencephalitis	Disorientation, exanthema		7 100% MNN	53	58	1.6	+	-
18	38/F	Meningoencephalitis	Fever, decreased consciousness.		261	173	67	4.6	+	-
19	16/M	Meningoencephalitis	Exanthema, headache, fever, vomiting, disorientation		13 100% MNN	91	79	2.5	+	-
20	32/F	Meningoencephalitis	Headache, exanthema, neck stiffness, disorientation		112 92% MNN 8% PMN	434	73	2.6	-	DENV-2
21	9/M	Meningitis	Headache, vomiting		202 100% MNN	66	67	2.1	+	-
22	40/M	Meningitis	Headache, myalgia		25 100% MNN	37.3	95	2.2	+	CMV
23	9/F	Meningitis	Headache, fever		384 100% MNN	66	50	1.4	+	-
24	25F	Meningitis	Headache, fever, neck stiffness		277 100% MNN	47	48	1.5	+	-
25	15/F	Meningitis	Exanthema, headache, fever		13 100% MNN	19	48	1.8	+	-
26	26/F	Meningitis	Headache		320 100% MNN	75	65	1.0	-	DENV-1
27	17/F	Acute myelitis	n.a		0	18	62	1.1	+	-
28	21/M	Encephalomyelitis	Headache, neck stiffness, exanthema		32 98% MNN 2% PMN	464	14	4.3	+	-

WBC: white blood cells; MNN: mononuclear cells; PMN: polymorphonuclear cells; CSF: cerebrospinal fluid; PCR: polymerase chain reaction; IgM: immunoglobulin M; “+” = positive; “-” = negative; MT: *Mycobacterium tuberculosis*. “n.a.” = not available.

**Fig. 2:**
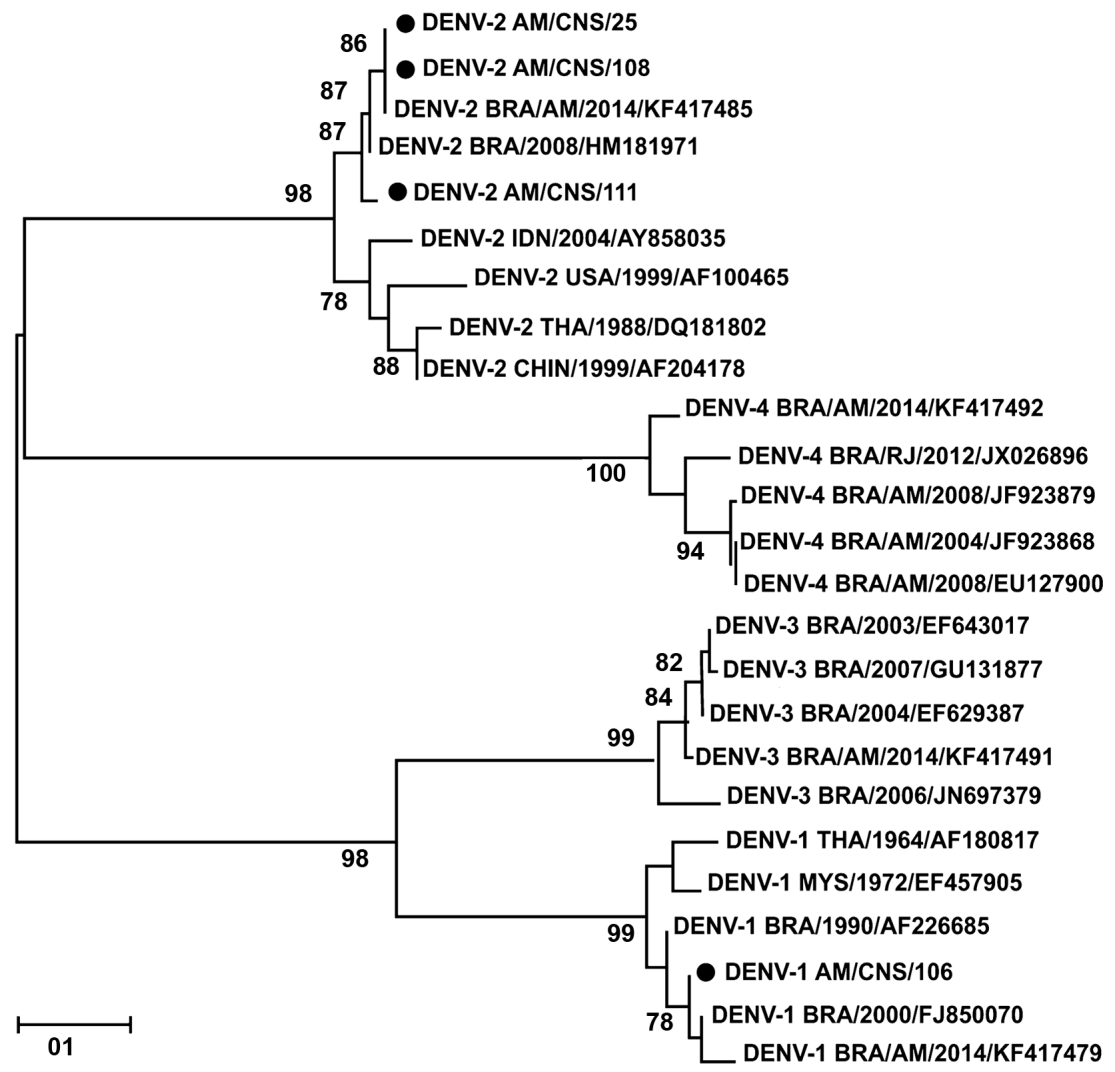
phylogenetic tree of dengue virus (DENV) isolated from cerebrospinal fluid (CSF) of patients with viral infection of central nervous system (CNS). The neighbour-joining tree was constructed using MEGA-X Software based on the prM-C genomic region and sequences were compared to sequences retrieved from Gene-Bank (NCBI, USA). Sequences from this study are marked with diamonds. Only boot-strap values above 75% are shown in the figure.

Of the 28 patients included in the study, 10 patients (35.7%, 10/28) were diagnosed with either hypoglycorrhachia (< 2/3 of serum glycaemia) or high levels of lactate (> 2.8 mmol/L) in the CSF. Among these ten patients, five were case of encephalitis, four were case of meningoencephalitis, and one case was that of encephalomyelitis.

DENV IgM was detected in CSF of 24 patients (85.7%, 24/28) by ELISA while infection in remaining four patients was confirmed by molecular typing (RT-PCR). The DENV serotypes for these four were determined by amplification and sequencing followed by sequence analysis of a 453 base pairs fragment of DNA from the prM-C region of DENV genome. Of these four CSF samples analysed, one case was of meningitis with DENV-1 serotype infection and three cases that developed encephalitis, meningitis, and meningoencephalitis were infected with DENV-2 serotype. Nucleotide sequences were aligned and neighbour-joining phylogenetic trees were generated for DENV serotype, as shown in Fig. 2.

*Co-infection with other agents* - Eight patients (28.6%, 8/28) had co-infection. Patient 22 was diagnosed with meningitis and had CMV and DENV co-infection in the CSF. Remaining seven (25.0%, 7/28) patients had HIV and DENV co-infection. Of these, five were diagnosed as encephalitis and two were diagnosed as meningoencephalitis. In addition to HIV and DENV, Patient 1 (encephalitis) was also infected with EBV and Patient 10 (meningoencephalitis) was infected with *Mycobacterium tuberculosis.* The CSF was inflammatory in all except for one case (Patient 8). It was characterized by median WBC count of 133 cells/mm^3^ (range 0 to 2048), median protein level of 121.5 mg/dL (range 27 to 483), median glucose level of 48.5 mg/dL (range 34 to 160), and median lactate level of 3.4 mmol/L (range 1.4 to 6.3) (Table).

## DISCUSSION

Neurological complications of DENV infection have been reported since 1976.^19^ Some of these complications, such as encephalitis, meningitis, and myelitis, may occur immediately after the first day of onset of systemic symptoms or after oligosymptomatic dengue infection. The presence of viral RNA is not routinely detected during the course of neurological manifestations associated with dengue. Therefore, in addition the detection of IgM antibodies in serum/CSF may also be carried out for the confirmation of diagnosis.

We highlight the importance of routine molecular and immunological analysis of CSF for the diagnosis of DENV infection of CNS. We analysed CSF samples from 700 patients living in dengue-endemic area (Amazonian Region) who were suspected of acute CNS viral infection and were treated at a reference centre of tropical medicine. DENV infection was detected in CSF samples of 30 (4.3%) patients. The presence of DENV RNA was verified in only four cases (DENV-1 or DENV-2). Eight patients with DENV were also co-infected with either CMV (one case) or HIV (seven cases). Two of patient’s with HIV co-infection were also infected with either EBV, or *M. tuberculosis* each. We found pleocytosis, hyperproteinorrachia, and hypoglycorrhachia / increased lactate in the majority (89%) of the patients with DENV infection in the CSF. A case of DENV, HIV, and *M. tuberculosis* co-infections had a very marked pleocytosis (2048 cells/mm^3^), which is reported to occur in less than 9% of the tuberculous meningitis.^9^ Another patient with meningoencephalitis associated with DENV also showed very high pleocytosis (1226 cells/mm^3^).

The glucose in CSF is derived from the plasma. Low levels of CSF glucose may be masked by hyperglycaemia, such as in the case of Patient 9. The level of lactate in CSF is not dependent on blood, but produced in the brain. In addition to estimation of glucose levels, the lactate level analysis also contributed to the accuracy of the CSF examination. Increased CSF lactate levels and lower amount glucose is also seen in case of bacterial meningitis and, are also normal occurrences in viral CNS infection. Results of CSF examination are usually normal but may some time show discrete abnormalities in neurological disorders associated with dengue infection.^20^ ^,^ ^21^ ^,^ ^22^ Increased detection of inflammatory CSF observed in our study compared to other studies may be justified by the fact that severe acute cases of patients visit the emergency department of our reference centre of infectious diseases (FMT-HVD) in Amazonian. It also may be due comorbidity with other infectious agents, or due the immunogenetic background of the Amazonian population associated with indigenous ancestry.

In the current analysis, the most frequent neurological manifestation was encephalitis followed by meningoencephalitis, meningitis, encephalomyelitis, and acute myelitis. These findings are in accordance with other studies.^22^ The frequency of neurological disorders associated with DENV varies throughout the world (0.5-21%).^20^ It depends on the type of study, the techniques, and the samples used for laboratory diagnosis. Moreover, specific CSF test results have not been frequently reported. In an earlier study from Vietnam, 27 (0.5%) children with encephalopathy out of 5,400 patients with dengue haemorrhagic fever were confirmed by RT-PCR and IgM antibodies in serum/CSF.^21^ Also, in a previous report from Brazil, of the 85 cases of acute dengue infection confirmed by multiplex-RT-PCR/Real-Time PCR of CSF/serum samples it was demonstrated that 18 (21.2%) patients had neurological involvement.^22^ Araujo et al.^24^ found that 3.8% (8/209) of CSF tested positive for dengue in a study of 183 patients with suspected viral meningitis/meningoencephalitis and 26 deceased patients with suspected meningitis^24^. Marinho et al.^25^ detected DENV-1, -2, and -3 co-infections in 31.8% (7/22) of CSF of paediatric patients having viral meningitis.^24^ ^,^ ^25^.

A previous study of 165 suspected of viral CNS infection, in the Amazonian Region (Brazil), by RT-PCR, four cases (2.4%) tested positive for DENV in CSF.^26^ The present study reports an improvement in the accuracy of diagnosis (4.3%) in the same region by the combined use of DENV RT-PCR and IgM ELISA test of the CSF.

CSF analysis is critical and may be quite helpful in establishing aetiology in CNS infection. Demonstration of specific viral IgM antibodies in CSF from patients with neurological disorders caused by numerous viruses is considered to be the diagnostic test of choice for neuroinvasive disease.^11^ Detection of dengue IgM in CSF has shown high specificity (97%), but limited sensitivity (46-73%), for neurological conditions.^27^. In our study, we detected IgM antibodies in CSF from 87% of the confirmed cases of dengue CNS infection using an in-house ELISA test already adapted for CSF analysis.^10^ In addition, the utility of nucleic acid amplification testing by PCR of CSF samples has greatly increased the ability to diagnose infections of the CNS, especially viral infections caused by different agents.^26^ ^,^ ^28^ However, dengue RNA is detectable only during the viraemia period (i.e. during the first week of symptoms onset). Therefore, it is not detected in the CSF regularly. We demonstrated the presence of viral RNA in 13% (4/30) of our cases. Albeit, it is important to note that the combination of different diagnostic methods contribute towards increased identification of the agents responsible for CNS infections.^22^

Since its introduction in 1998, Manaus has recorded the three large outbreaks of DENV. All four serotypes are disseminated throughout the region and are considered endemic.^5^ ^,^ ^7^ ^,^ ^29^ Nucleotide sequencing of the selected samples were performed to identify whether the DENV genotype circulating in the nervous system was similar to the local virus causing febrile disease. We observed the presence of DENV-1 and DENV-2 serotypes. The DENV-1 and DENV-2 serotypes belong to genotype V and Asian/American, respectively. DENV-2, Asian/American, is the more virulent genotype. Earlier report by Martins et al.^7^ did not observe an association between DENV-1 genotype V and severe disease, but here we found a case associated with severe dengue meningitis caused by genotype V. A previous study also has demonstrated a severe neurological disorder associated with DENV-1.^6^ A recent analysis in the paediatric population has shown an association between the Asian II genotype of DENV-2 and meningitis, but in our current study we detected only DENV-2 of Asian/American genotype causing severe dengue in CNS (encephalitis and meningoencephalitis).^25^

The Brazilian population has a heterogeneous ethnic composition, resulting from the mixture between indigenous native populations and immigrants from Europe, Africa, and Asia. The literature shows that the flow of immigration was not uniform in the different regions of Brazil.^30^ ^,^ ^31^ The Amerindian contribution was higher in the north of the country, while European migration occurred more in the south and southeast regions. The variation in the ethnic composition of the urban and rural populations of the Brazilian Amazon and distinct regions in Brazil may influence the genetic mechanisms favourable to susceptibility to infectious and parasitic diseases and co-infections.^31^

In relation to the clinical symptoms, dengue infection is usually asymptomatic. Clinical manifestations may range from fever to forms of severe dengue, such as neurological complications. Mild forms include fever, headache, malaise, nausea, vomiting, myalgia, arthralgia, and maculopapular rash. It can last up to seven days. Neurological manifestations occur between two and 30 days after the onset of the febrile period, at different stages of the disease, and sometimes without any signs and symptoms of dengue infection. These data justify the reduced frequency of signs and symptoms for acute dengue observed in our cases. Therefore, the percentage of patients who presented with fever was much lower (39.5%) than that has been previously described in the literature for dengue cases (> 80%).^23^ The limitation of this study was the time between the onset of symptoms and the date of collection of the CSF samples. Generally, patients seek medical assistance when the symptoms are severe. By this time, the virus is sometimes not detectable in the CSF. This can also contribute to the lower detection viral genome. It is therefore important to investigate for specific IgM antibodies that last longer in CSF. This study was conducted retrospectively; therefore, serum samples were not available for paired analysis along with CSF samples. Hence, it may be possible that the frequency of neurological cases associated with dengue was underestimated, considering the limited sensitivity of DENV IgM detection in CSF.

In conclusion, our findings of acute inflammation associated with DENV detection in CSF confirm the disease in CNS and viral neurotropism. In general, the routine CSF analysis may be able to clinically discriminate between viral and bacterial CNS infection. However, the combined epidemiological, clinical, and laboratory approaches contribute to DENV diagnosis in the majority of cases. The dengue viral identification (immunological and molecular) is also an important tool for epidemiological data. In addition, these findings suggest public health officials should investigate dengue as a cause of acute viral encephalitis, myelitis, and meningitis in endemic areas as well as the immunity of indigenous native populations against endemic pathogens such as DENV.
